# Silicate Fertilizer Amendment Alters Fungal Communities and Accelerates Soil Organic Matter Decomposition

**DOI:** 10.3389/fmicb.2019.02950

**Published:** 2019-12-20

**Authors:** Suvendu Das, Jeong Gu Lee, Song Rae Cho, Hyeon Ji Song, Pil Joo Kim

**Affiliations:** ^1^Institute of Agriculture and Life Sciences, Gyeongsang National University, Jinju, South Korea; ^2^Division of Applied Life Sciences, Gyeongsang National University, Jinju, South Korea

**Keywords:** slag silicate fertilization, fungal communities, soil enzyme activities, illumina sequencing, rice paddy

## Abstract

Soil microorganisms play a crucial role in organic matter decomposition and nutrient cycling in cropping systems. Compared to bacteria, fungal community composition and the role of fungi in organic matter decomposition and nutrient cycling in agro-systems are, however, elusive. Silicon (Si) fertilization is essential to improve agronomic performance of rice. The effects of the Si fertilizer application on the soil fungal community composition and their contribution in soil organic matter (SOM) decomposition are not yet studied. We investigated the short-term (120 days) slag silicate fertilizer (SSF) amendment impacts on plant photosynthesis and soil biochemical changes, soil fungal communities (assessed by ITS amplicon illumina sequencing), hydrolytic and oxidase enzyme activities, CO_2_ emissions, and bacterial and fungal respiration in diverse eco-geographic races of rice (*Oryza sativa* L.), i.e., Japonica rice (*O. sativa japonica*) and Indica rice (*O. sativa indica*). The short-term SSF amendment significantly increased the relative abundance of saprotrophic fungi and accelerated organic matter decomposition. The increase in saprotrophic fungi was mostly attributed to greater labile C availability and Si availability. Higher organic matter decomposition was accompanied by an increase in both hydrolytic and oxidative enzyme activities in response to the SSF amendment. The stimulation of oxidative enzyme activities was explained by an increase in root oxidase activities and iron redox cycling, whereas stimulation of hydrolytic enzyme activities was explained by the greater labile C availability under SSF fertilization. We conclude that the short-term SSF amendment increases saprotrophic fungal communities and soil hydrolytic and oxidative enzyme activities, which in turn stimulates SOM mineralization and thus could have negative feedback impacts on soil C storage in submerged rice paddies.

## Introduction

One of the central sustainability challenges for intensive agriculture is to increase the yield while decreasing environmental degradation. Rice (*Oryza sativa* L.) is the major staple food consumed by over half the world population. Rice is a silicon (Si) accumulating plant, which can accumulate Si above 10% of shoot dry weight (Meharg and Meharg, 2015). Silicon (Si) is recognized as an agronomically essential element for rice cultivation. Intensive rice cultivation to meet the food requirements of the growing population continually decreases Si content of paddy soils and thus degrades the soil and decreases rice yield (Sasaki et al., 2014). The application of Si fertilizer is therefore highly recommended for Si-poor soils for optimum plant performance and crop yield improvement. Further, with the rapid growth in iron/steel manufacture, the larger quantity of slag produced draws notice for the need for its use in an efficient way, such as in agriculture. The use of slag in agriculture not only mitigates environmental consequences raised due to the disposal of slag at landfill sites but also improves crop productivity (Gwon et al., 2018; Das et al., 2019b). Steel making slag has been commonly used in slag-based silicate fertilizer (SSF) manufacturing (Ito, 2015). The SSF is rich in Si, Ca, P, Mg, Fe, and Mn, but plant essential nutrients such as, N, P, and K contents in the SSF are low (Ito, 2015). It is therefore advisable to use the SSF along with a mineral/organic fertilizer that has significant amounts of N, P, and K. The SSF is generally applied to the soil to provide the Si benefits to the plant. Silicon preferentially deposited in plant epidermal tissues and improves tissue rigidity and increases biotic and abiotic stress resistance, thereby enhances plant growth and yield (Meharg and Meharg, 2015). However, due to its liming nature (rich in CaO content), the long-term SSF amendment could make the soil more alkaline, which in turn stimulate soil organic matter (SOM) decomposition (Castro et al., 2015). The application of SSF is, therefore, proposed to practice in interruption, when the soil Si content and the soil pH (<6.5) become low (Ali et al., 2009). In this context, evaluating the short-term effects of the SSF amendment in rice paddies would be informative.

Soil microorganisms are crucial in the functioning of healthy and productive ecosystems, playing a pivotal role in SOM degradation, nutrient acquisition, and plant immune response (van Wees et al., 2008; Jacoby et al., 2017). Changes in soil properties in response to soil amendments are linked to the shift in soil microbial communities that regulate ecosystem functions and plant growth and productivity (Jacoby et al., 2017). Like bacteria, fungi were also crucial for agricultural systems where they play vital roles in SOM decomposition, nutrient cycling, and plant growth (de Vries and Caruso, 2016). Moreover, saprotrophic fungi have a greater role to degrade the plant litter because of their relatively high efficiency to degrade lignocelluloses, and mycorrhizal fungi play a crucial role in plant growth by nutrient exchange in a symbiotic relationship (de Vries and Caruso, 2016). Compared to bacteria, the changes in fungal communities and their role in response to agricultural management practices are, however, less studied.

The SSF amendment may alter soil fungal diversity and increase fungal richness. This is based on the fact that adequate Si supply increases plant photosynthesis and above ground biomass (Detmann et al., 2012), which in turn increases below-ground C allocation through root exudation and likely increases soil microorganisms, including fungi. Moreover, the increased root C availability and root biomass in response to the SSF amendment could increase colonization by mycorrhizal fungi, and adequate rhizodeposit C could allow more fungal taxa to persist, increasing fungal diversity (de Vries and Caruso, 2016). The increase in below-ground labile C (easily decomposed root litter and exudates) likely alleviates C limitation and could allow more saprophytic fungi to colonize (de Vries and Caruso, 2016), which in turn could increase SOM decomposition under the SSF amendment. Further, as a rich source of iron, the SSF amendment in paddy soils may enhance iron redox transformation, which in turn may increase soil oxidative and hydrolytic enzyme activities and thus organic matter decomposition (Van Bodegom et al., 2005; Hall and Silver, 2013). There is evidence that Si not only increases plants’ resistance against pathogens, but also decreases the prevalence of pathogenic fungi albeit the underlying mechanism is not known (Wang et al., 2017). Silicon application is a preventive measure against a number of fungal diseases (Wang et al., 2017). It is therefore assumed that as a rich source of Si, the SSF application could decrease the abundance of pathogenic fungi. Based on these reasons, we hypothesize that the SSF amendment would (i) increase plant photosynthesis and C, Si, and Fe availability in soil, (ii) alter the soil fungal community structure and increase the relative abundance of saprophytic fungi, driven by higher labile C and Si availability, and (iii) enhance soil C degradation in terms of increased soil C fluxes, due to increasing hydrolytic (driven by greater labile carbon) and oxidative (driven by root oxidase activity and iron redox cycling) enzyme activities. To test the hypothesis, we evaluated the short-term SSF amendment effects on plant photosynthesis and soil biochemical changes, soil fungal community structure, hydrolytic and oxidase enzyme activities, CO_2_ emissions, and bacterial and fungal respiration in Japonica and Indica rice varieties. Multivariate analyses were conducted to link fungal community structure to soil and plant variables, soil enzyme activities, and CO_2_ emissions.

## Materials and Methods

### Experimental Design

The greenhouse pot experiment was carried out at Gyeongsang National University (36°51′N, 128°28′E), Jinju, South Korea. The soil used for the pot experiment was collected from the nearby paddy field that has not been amended with any fertilizer and/or manure. The soil was air dried, crushed, sieved (0.5 cm sieve), homogenized and 14 kg soil was packed in Wagner pots (0.24 m diameter and 0.3 m height) to bulk density of 1.2 g cm^–3^. The soil was fine-silty, mixed, mesic family of Typic Endoaquepts and have the following properties: pH (1:5 soil:water extract) 5.6, total soil organic carbon (TOC) 25.8 g kg^–1^, total nitrogen (TN) 2.76 g kg^–1^, available phosphorous (AP) 0.76 g kg^–1^, and available Si 0.86 g kg^–1^. There were four treatments: (1) Japonica rice cultivated without SSF (JC), (2) Japonica rice cultivated with SSF (JS), (3) Indica rice cultivated without SSF (IC), and (4) Indica rice cultivated with SSF (IS). Two major eco-geographic variety of rice, i.e., Japonica (*cv* Dongjinbyeo) and Indica (*cv*Rc158) with the equal growth duration (120 days) were selected for the pot experiment. Rice seedling (25 days old) were transplanted with two seedlings per pot. The soil was flooded with water 3 days before transplanting. The water level of all pots was retained at about 5 cm above the soil surface during the entire cropping season. The mineral fertilizer was applied to all the pots at the rate of N-P_2_O_5_-K_2_O = 110-45-58 kg ha^–1^ by using urea, fused superphosphate, and potassium chloride (Das et al., 2019a). Basal fertilizers broadcasted 1 day prior to transplanting were 55 kg N ha^–1^, 45 kg P_2_O_5_ ha^–1^, and 40 kg K_2_O ha^–1^, while 22 kg N ha^–1^ and 18 kg K_2_O ha^–1^ were broadcasted at the mid vegetative (tillering) stage, and 33 kg N ha^–1^ was broadcasted at the early reproductive (panicle initiation) stage. For SSF treatments, the SSF was amended at the rate of 2 Mg ha^–1^, 3 days prior to transplanting. The chemical composition of the SSF was provided in Supplementary Table S1. All chemical and SSF fertilizers were applied according to the Rural Development Administration farm management practice guideline for rice cultivation in South Korea (Rural Development Administration [RDA], 2010). Experiments were performed in triplicate.

### Sampling and Analysis

Soil samples were collected at different growth stages of the crop, i.e., seedling (1–20 days), tillering (15–20 days), panicle initiation (15–20 days), heading (30 days), and ripening (30 days) for a 120 days rice crop. Soil redox potential (Eh) was measured using Eh meter (PRN-41, DKK-TOA CORPORATION, Tokyo) and soil pH was measured by a pH meter (Orion 3 star, Thermo Electron Corporation, United States). The readily mineralizable carbon (RMC) and nitrogen (Ninhydrin nitrogen, NRN) content of soil were estimated by the method previously described by Das and Adhya (2014). The SOC content was estimated at the harvesting stage by a dry combustion C/N analyzer (CHNS-932 Elemental Analyzer, Leco, United States). The photosynthetic rate of tagged leaf was estimated using a photosynthesis apparatus (CIRAS-2, PP-Systems, United Kingdom) during 9:00 am to 10:00 am. Separate planted pots from each of the treatments were sacrificed for the analysis of root oxidase activity. The root oxidase activity was measured by α-Naphthylamine oxidation method (Gutierrez et al., 2014). The soil pore-water was sampled using rhizo-samplers (EcoTech^®^, Germany) and collected in acid washed, N_2_ flushed, crimp sealed vials. Silicon (aqSi) and Fe (aqFe) in soil pore-water were quantified using inductively coupled plasma-optical emission spectrometry (ICP-OES) (Vista-MPX, Varian, Australia).

### Molecular Characterization of Fungi

Microbial DNA was extracted from 0.5 g of freshly collected rhizosphere soil using a FastDNA SPIN kit for soil (MP Biomedicals, United States) following the manufacturer’s guideline. For each treatment, DNA was extracted in triplicate and pooled to minimize extraction bias, and quantified using NanoDrop 1000 spectrophotometer (NanoDrop Technologies, Wilmington, DE, United States). PCR amplification was performed in a total volume of 50 μL reaction mixture containing 10 ng of template DNA, 10 × AccuPrime PCR buffer II (including dNTPs) (Invitrogen, United States), 0.2 U AccuPrime High Fidelity Taq Polymerase, and 0.4 mM of both forward and reverse primers. The primer pair used to amplify fungi was ITS3/ITS4 (Li et al., 2016). The paired Illumina adaptors were linked to the 5′ end of the forward and reverse primers. The PCR conditions to amplify fungal ribosomal internal transcribed spacers (ITS) consisted of an initial denaturation at 95°C for 8 min, 36 cycles of denaturation at 95°C for 45 s, annealing at 58°C for 45 s and elongation at 72°C for 1 min followed by a final elongation at 72°C for 8 min. Each sample was amplified in triplicates, pooled, gel purified using AxyPrep DNA Gel Extraction Kit (Axygen Biosciences, CA, United States), and quantified. To reduce PCR bias and to minimize PCR drift, three replicates PCR amplification was conducted. The amplicons were combined in equimolar amounts and paired-end sequenced (2 × 250) on an Illumina MiSeq platform (Illumina Inc., San Diego, CA, United States). Low quality reads (i.e., reads with any ambiguous bases, mismatches to primers, homopolymers longer than 8 bp, or less than 300 bp in length) were removed using QIIME pipeline (Caporaso et al., 2012). Processed sequences with an identity threshold of 97% were clustered into OTUs. Taxonomy was assigned using the BLAST algorithm against the UNITE database (Li et al., 2016). Alpha diversity metrics were calculated based on rarefied OTU tables in QIIME. In addition, the OTUs were assigned to the fungal functional guild (mostly saprotrophs, pathotrophs, and symbiotrophs) using the FUNGuild annotation tool (Nguyen et al., 2016). Sequences were uploaded to the National Center for Biotechnology Information (NCBI) Sequence Read Archive under BioProject PRJNA491385.

### Soil Enzyme Assays

Freshly collected rhizosphere soil samples at the harvesting stages of rice were used to study soil enzyme activities. The plant was uprooted from the pot, shaken to remove excess soils on roots, and soils adhering to roots were washed with sterile distilled water and centrifuged at 10000 × *g* for 10 min to collect rhizosphere soils (Yergeau et al., 2014). The activities of hydrolytic enzymes [α-glucosidase (EC 3.2.1.20), β-1,4-glucosidase (EC 3.2.1.21), β-1,4-xylosidase (EC 3.2.1.37), and cellobiohydrolase (CBH; EC 3.2.1.91)] were measured by the microplate flurometric method, whereas oxidase enzyme [Phenol oxidase (EC 1.10.3.2) and peroxidase (EC 1.11.1.7)] activities were colorimetrically estimated using 96-well microplate according to the methods reported by Liu et al. (2017).

### CO_2_ Emissions and C Mineralization Using Selective Inhibitors

Carbon dioxide emission fluxes during the rice cropping period were estimated following the close static chamber method as described by Gwon et al. (2018). The CO_2_ production from the soil (0–15 cm depth) was estimated by placing the moist soil sample (20 g) in a sterile serum bottle (volume = 120 mL). The bottle was closed with neoprene septa, crimp-sealed, and incubated at 28°C in the dark. The CO_2_ production was estimated at every 3 days interval for 3 weeks by a gas chromatography (Shimadzu, GC-2010, Japan) equipped with a thermal conductivity detector (Gwon et al., 2018). The contribution of fungal or bacterial decomposition (CO_2_ production) was determined in soil incubation experiments by selective inhibition with added Streptomycin sulfate (a bactericide) or Cycloheximide (a fungicide) (Ananyeva et al., 2010; Seo and DeLaune, 2010). Streptomycin sulfate was added to soils at 3.0 mg g^–1^, while Cycloheximide was added to soils at 1.5 mg g^–1^. Optimal inhibitor concentrations were determined using a preliminary screening experiment based on the criteria of Anderson and Domsch (1975). Additional details in methodology are given in the Supplementary Material.

### Statistical Analysis

The treatment effects (SSF and cultivar) on the measured parameters were tested using two-way analyses of variance (ANOVA) according to the general linear model. The *Post hoc* comparison was performed by Tukey’s honestly significant difference (HSD) test. The difference in fungal community composition (Bray–Curtis dissimilarities) was assessed by permutational multivariate analysis of variances (PERMANOVA). To demonstrate the changes in relative abundance of fungi, the response ratio (RR) was estimated and to determine whether the RR was significantly different from zero, one-sample *t*-test was conducted. The relationship between the soil fungal community and soil/plant variables, soil enzyme activities, and CO_2_ emissions were assessed by conducting mantel test, and canonical correspondence analysis (CCA). The analyses were conducted using the vegan package (v. 1.15-1) of R software (v. 2.8.1) (Oksanen et al., 2013).

## Results

### Soil Biochemical Properties and Photosynthetic Rate

The SSF application significantly (*P* < 0.05) increased soil pH, whereas soil Eh was not significantly influenced in both Japonica and Indica rice (Supplementary Figure S1 and Supplementary Table S2). The soil RMC content was significantly (*P* < 0.05) increased, but the soil NRN content was significantly (*P* < 0.05) decreased at maximum tillering (42 DAT), heading (75 DAT), and harvesting (120 DAT) stages of rice, in response to the SSF application (Supplementary Figure S1). On an average, 40.5 and 44.0% increase in soil RMC content, 15.3 and 15.8% decrease in soil NRN content in Japonica and Indica rice, respectively, were observed. The plant photosynthetic rate and root oxidase activity were significantly (*P* < 0.05) increased as a result of the SSF amendment in different plant growth stages of rice, irrespective of cultivar variation (Supplementary Figure S1). The photosynthetic rate was increased by 25.4 and 27.7% and the root oxidase activity was increased by 19.2 and 23.0% in Japonica and Indica rice. In comparison to no amended control, the SSF amendment significantly (*P* < 0.05) increased aqSi by 515 and 443%, and aqFe by 39.6 and 76.7% in Japonica and Indica rice, respectively (Supplementary Figure S1 and Supplementary Table S2). The grain yield, straw biomass, and root biomass were significantly (*P* < 0.05) increased by 17.9 and 15.3%, 25.1 and 36.8%, and 10.9 and 15.3% in the SSF amended Japonica and Indica cultivars, respectively, corresponding to no amended control.

### Fungal Community Diversity

**FIGURE 1:**
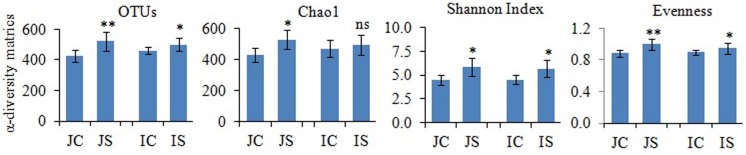
The slag silicate fertilizer (SSF) effects on fungal α-diversity indices. Significant difference between control and SSF within different cultivars were tested by two-tailed paired *t*-tests as indicated by ^∗^ when *P* < 0.05 and ^∗∗^ when *P* < 0.01. JC, Japonica rice cultivated without SSF; JS, Japonica rice cultivated with SSF; IC, Indica rice cultivated without SSF; IS, Indica rice cultivated with SSF.

Rarefaction curves of the observed species and Chao1 were used to assess whether the sequencing depth of the samples was adequate to signify the fungal communities in paddy soils. All rarefaction curves are quite smooth (Supplementary Figure S2), which showed that the sequencing depth adequately covers the diversity (Li et al., 2016). Further, Good’s coverage was high on average (mean = 0.99) indicating that fungal communities were sufficiently sampled. The number of reads sequenced from soil samples ranged from 151052 to 174372 (Supplementary Table S3). The proportion of high-quality reads ranged from 95.21 to 95.612% (Supplementary Table S3). The number of OTUs ranged from 426 to 521, and significantly (*P* < 0.05) increased by the SSF amendment (Figure 1). The number of fungal OTUs observed in our study was more similar to the fungal OTUs observed in other submerged paddy ecosystems (Yuan et al., 2018; Zhang et al., 2019).

**FIGURE 2:**
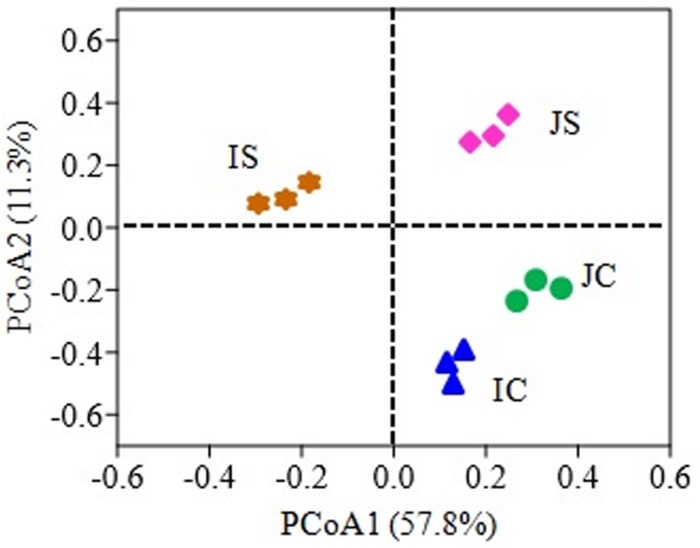
Principal coordinate analysis (PCoA) plot based on the fungal OTUs for Japonica and Indica rice cultivated with and without slag silicate fertilizer (SSF). JC, Japonica rice cultivated without SSF; JS, Japonica rice cultivated with SSF; IC, Indica rice cultivated without SSF; IS, Indica rice cultivated with SSF.

The SSF amendment significantly (*P* < 0.5) increased fungal species richness (Chao1) and diversity (Shannon index) compared to the unamended control in both Japonica and Indica rice (Figure 1). However, no significant variation of the measured diversity indices within cultivars was found. Principal coordinate analysis (PCoA) based on fungal OTUs showed a clear difference in the fungal community structure between SSF amended and unamended treatments, irrespective of the rice cultivar (Figure 2). The first two principal coordinates explained 69.1% of the total variance of the fungal community.

**FIGURE 3:**
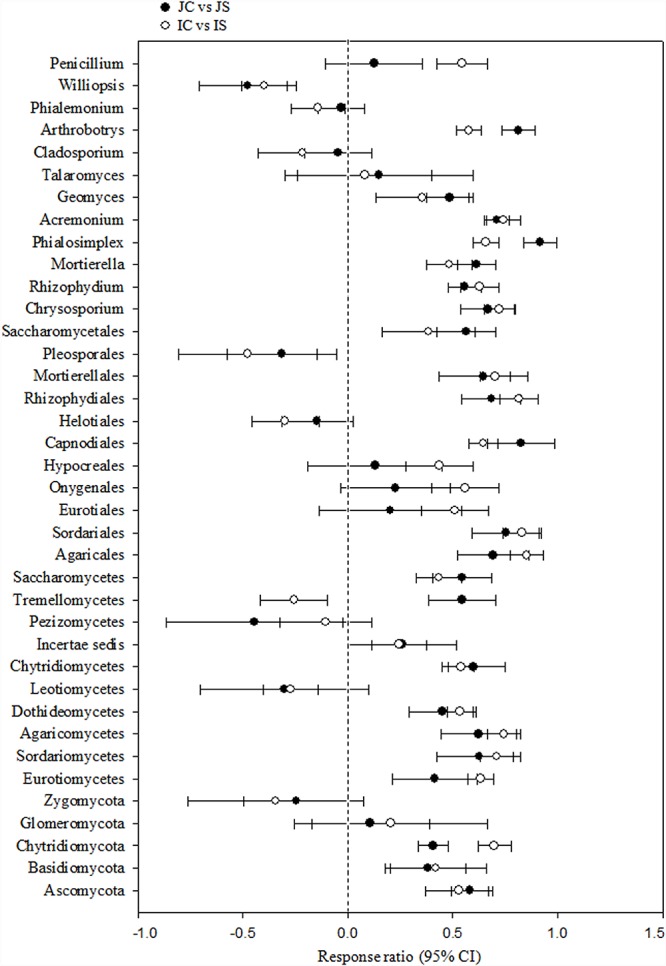
The response ratio of major fungal lineage at 95% confidence interval (CI). JC, Japonica rice cultivated without SSF; JS, Japonica rice cultivated with SSF; IC, Indica rice cultivated without SSF; IS, Indica rice cultivated with SSF.

**FIGURE 4:**
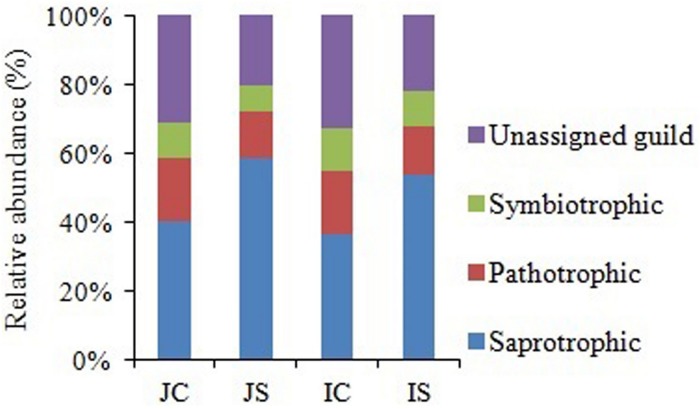
The relative abundance of fungal functional guild in Japonica and Indica rice in response to the SSF amendment. JC, Japonica rice cultivated without SSF; JS, Japonica rice cultivated with SSF; IC, Indica rice cultivated without SSF; IS, Indica rice cultivated with SSF.

Soil fungal communities were dominated by phylum Ascomycota (4.6–15.6%) in all the treatments. Next to Ascomycota, the other two abundant phyla were Basidiomycota (1.0–2.2%), and Chytridiomycota (0.17–0.92%). All the three phyla were significantly increased by the SSF application, regardless of the rice cultivar (Figure 3 and Supplementary Table S4). Eurotiomycetes (1.3–5.6%), Sordariomycetes (1.3–4.0%), Agaricomycetes (1.0–2.1%), Dothideomycetes (0.4–2.0%), Leotiomycetes (0.2–4.3%), and Chytridiomycetes (0.2–1.0%) were the five most abundant classes, whereas Agaricales (0.9–1.9%), Sordariales (0.5–2.1%), Eurotiales (0.7–2.9%), Onygenales (0.7–2.7%), and Hypocreales (0.6–1.7%) were the five most abundant orders in all sequences. The SSF amendment significantly increased most of the abundant fungal classes and orders in both the rice cultivars (Supplementary Table S4). Among the most abundant genera, *Chrysosporium*, *Rhizophydium*, *Mortierella*, *Phialosimplex*, *Acremonium*, *Geomyces*, *Talaromyces*, *Arthrobotrys*, and *Williopsis* were considerably (*P* < 0.05) greater in the SSF amended treatments compared to unamended treatments, irrespective of rice cultivars (Figure 3 and Supplementary Table S4). The analysis of the fungal functional ecological guild revealed that saprotrophs dominated in the paddy soil, followed by pathotrophs and symbiotrophs (Figure 4). The SSF amendment significantly increased the relative abundance of saprotrophic fungi (*P* < 0.01), whereas the shift in symbiotrophic and pathotrophic fungi were not significant.

### Soil Enzyme Activities

**FIGURE 5:**
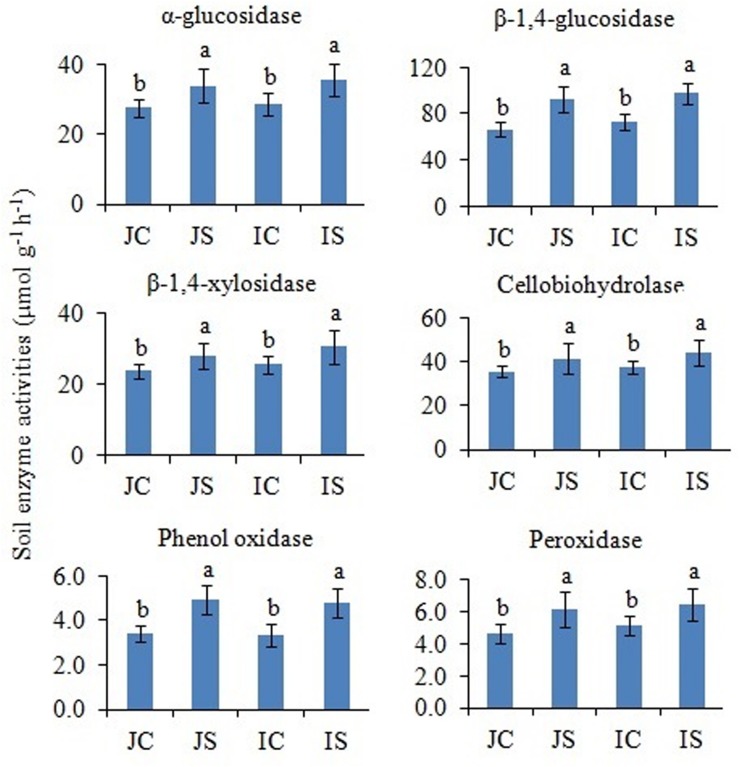
Effects of the SSF amendment on soil enzyme activities in Japonica and Indica rice. In bar diagrams, different letters within the treatments represent a significant difference (*P* < 0.05). JC, Japonica rice cultivated without SSF; JS, Japonica rice cultivated with SSF; IC, Indica rice cultivated without SSF; IS, Indica rice cultivated with SSF.

In comparison to unamended control, the SSF amendment significantly (*P* < 0.05) increased both hydrolytic (i.e., α-glucosidase, β-1,4-glucosidase, β-1,4-xylosidase, and cellobiohydrolase) and oxidase (i.e., phenol oxidase and peroxidase) enzyme activities, irrespective of the cultivar (Figure 5). The cultivar effect on the measured soil enzyme activities was not significant (Supplementary Table S2).

### SOC, CO_2_ Emissions, and Bacterial and Fungal Respiration

**FIGURE 6:**
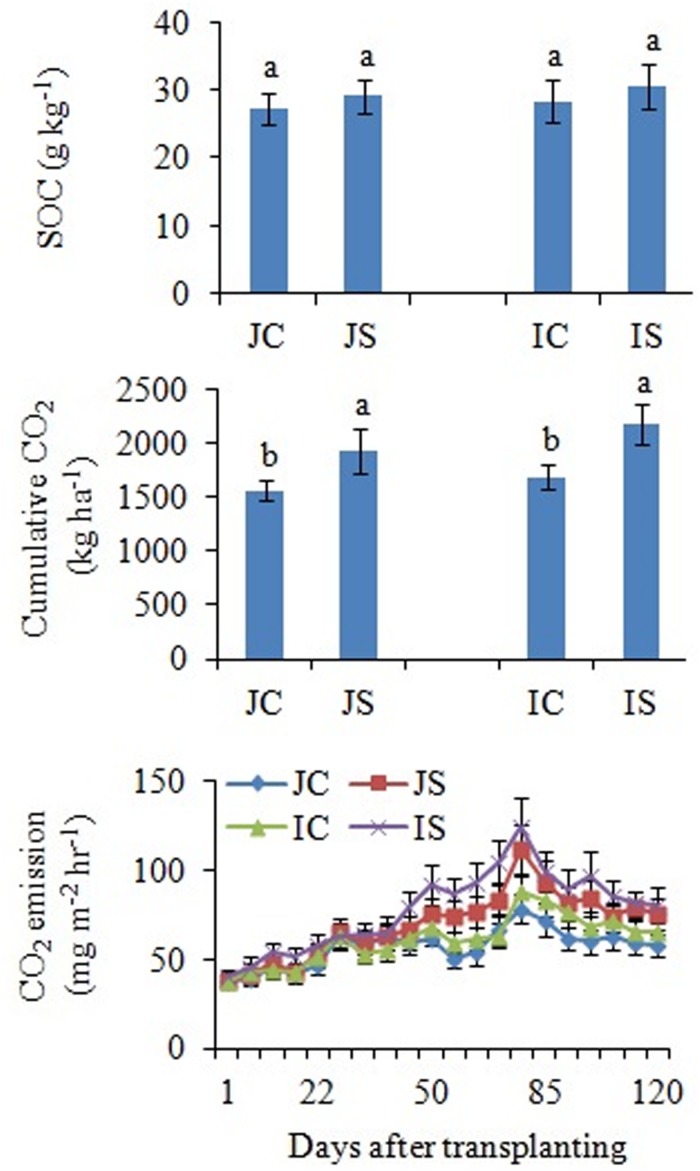
Effects of the SSF amendment on SOC content, cumulative CO_2_ emissions, and CO_2_ emission rate from Japonica and Indica rice. In bar diagrams, different letters within the treatments represent a significant difference (*P* < 0.05). JC, Japonica rice cultivated without SSF; JS, Japonica rice cultivated with SSF; IC, Indica rice cultivated without SSF; IS, Indica rice cultivated with SSF.

**FIGURE 7:**
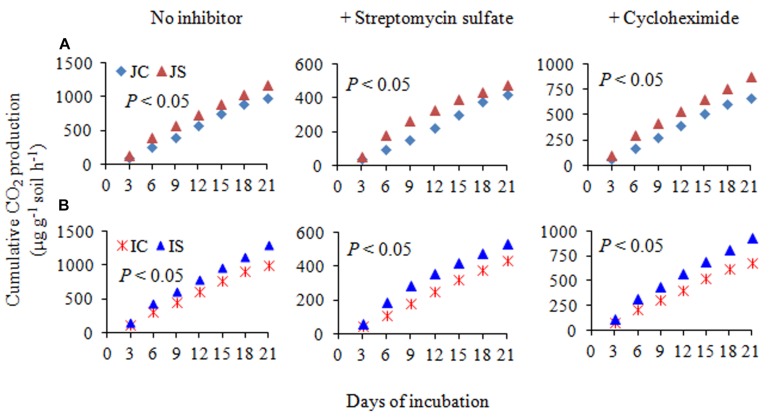
The cumulative CO_2_ production with no inhibitor, +streptomycin sulfate, and +cycloheximide in **(A)** Japonica and **(B)** Indica paddy soil. JC, Japonica rice cultivated without SSF; JS, Japonica rice cultivated with SSF; IC, Indica rice cultivated without SSF; IS, Indica rice cultivated with SSF. Bacterial and fungal cumulative CO_2_ production was estimated from the soil treated with cycloheximide and streptomycin sulfate, respectively.

The changes in SOC content in response to the SSF amendment were not significant, irrespective of the cultivar (Figure 6 and Supplementary Table S2). The SSF amendment significantly (*P* < 0.05) increased cumulative CO_2_ emissions by 23.2 and 29.0% (Figure 6), whereas the cumulative CO_2_ production was significantly (*P* < 0.05) increased by 25.5 and 29.4% in Japonica and Indica rice, respectively, in comparison to no amended control (Figure 7). The application of the bactericide Streptomycin sulfate resulted in about 57.0% lowering of the cumulative CO_2_ production, whereas the application of the fungicide Cycloheximide resulted in about 28% lowering of the cumulative CO_2_ production in comparison to the treatment to which no inhibitor had been added (Figure 7). The SSF amendment also significantly (*P* < 0.05) increased bacterial cumulative CO_2_ production (soil treated with Cycloheximide) by 36.3 and 39.0% and fungal cumulative CO_2_ production (soil treated with Streptomycin) by 30.8 and 33.3% in Japonica and Indica rice, in comparison to no amended control (Figure 7). The cultivar effects on CO_2_ emissions, cumulative CO_2_ production, and bacterial and fungal cumulative CO_2_ production were not significant (Supplementary Table S2).

### Linking Soil Fungal Communities to Soil and Plant Variables, Soil Enzyme Activities, CO_2_ Emissions, and Fungal CO_2_ Production

**FIGURE 8:**
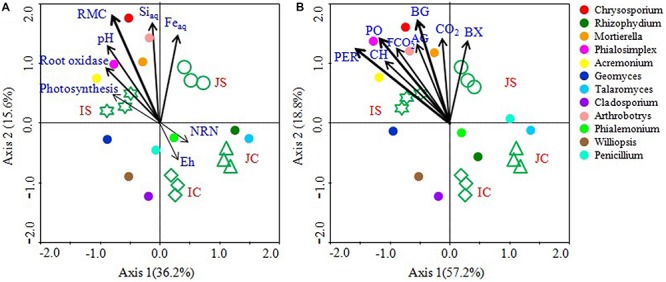
Canonical correspondence analysis (CCA) showing linkage of the top 12 fungal genera and soil and plant parameters **(A)** and soil enzyme activities, CO_2_ emission, and fungal CO_2_ production **(B)**. The lengths of these arrows indicate the relative importance of measured variables, whereas the angle between the arrows and the axis reflects the degree to which they are correlated. To statistically evaluate the significance (*P* < 0.01) of the first canonical axis and of all canonical axes together, the Monte Carlo permutation full model test with 999 unrestricted permutations was performed. RMC, Readily mineralizable carbon; NRN, Ninhydrin nitrogen; AG, α-glucosidase; BG, β-1,4-glucosidase; BX, β-1,4-xylosidase; CH, Cellobiohydrolase; PO, Phenol oxidase; PER, Peroxidase; CO_2_, CO_2_ emission; FCO_2_, Fungal CO_2_ production.

Canonical correspondence analysis analyses and mantel test revealed that the soil RMC content, aqSi, aqFe, soil pH, and root oxidase activity were significantly correlated with the fungal community (Figure 8 and Supplementary Table S6). Among the studied soil and plant factors, RMC (*r* = 0.293, *P* = 0.001) had the strongest positive correlation with the fungal community, followed by aqSi (*r* = 0.203, *P* = 0.010), aqFe (*r* = 0.168, *P* = 0.031), and soil pH (*r* = 0.152, *P* < 0.044), and root oxidase activity (*r* = 0.114, *P* = 0.054) (Figure 8 and Supplementary Table S6). CCA analysis further showed that hydrolytic and oxidative enzyme activities, CO_2_ emissions, and fungal CO_2_ production significantly and positively correlated with the fungal community (Figure 8 and Supplementary Table S6). Among the top 12 abundant fungal genera, *Chrysosporium*, *Rhizophydium*, *Mortierella*, *Phialosimplex*, *Acremonium*, and *Arthrobotrys* showed significant and positive correlation with RMC content, aqSi, aqFe, soil pH, root oxidase activity, soil enzyme activities, CO_2_ emissions, and fungal CO_2_ production (Figure 8).

## Discussion

In accordance with our hypothesis, the increased belowground carbon (e.g., RMC content) availability, aqSi and aqFe concentrations, and soil pH altered the soil fungal community structure in general, and increased the relative abundance of the saprotrophic fungal community in particular, in response to the SSF amendment. It is suggested that an increase in Si availability increases mesophyll conductance, which in turn improves plant photosynthesis (Detmann et al., 2012). As a rich source of Si, the SSF amendment significantly increased Si concentration in soil pore-water and thus plant photosynthesis. The increase in plant photosynthesis enhances root exudates which form continuous and highly dynamic C source and are the key component of the labile C pool in soils (Jacoby et al., 2017). The classical food web model postulates that increased labile C favors bacterial proliferation (de Vries and Caruso, 2016).

We found, the short-term (120 days) SSF amendment remarkably increased both bacterial and fungal richness and diversity in a submerged rice cropping system (Das et al., 2019a). In recent years, a large number of studies revealed that labile C not only help bacterial proliferation, but also the fungal proliferation at the same extent and saprotrophic fungi consume more labile C than previously suggested (de Vries and Caruso, 2016). In this study, the striking changes in fungal community composition were a significant increase in the most dominant classes Agaricales and Sordariales, in which the majority of the species are saprotrophic (Klaubauf et al., 2010), and the increase in the relative abundance of dominant genera *Chrysosporium*, *Rhizophydium*, *Mortierella*, *Phialosimplex*, *Acremonium*, and *Arthrobotrys* under SSF fertilization. The members of these genera mostly include fast growing saprotrophs that mainly utilize simple soluble carbon (Klaubauf et al., 2010; Voříšková et al., 2014; Anthony et al., 2017). The dominant species, *Chrysosporium keratinophilum*, *Mortierella polycephala*, *Agaricales* sp., *Helotiales* sp., *Phialemonium inflatum*, and *Aspergillus sydowii*, which are saprotrophic fungi and widely found in soil habitats, noticeably increased in response to the SSF amendment (Supplementary Table S5). Saprotrophic fungi (r-selected) proliferate in the presence of easily accessible C source and photosynthetic C allocation to roots can stimulate the proliferation of saprotrophic fungi (Anthony et al., 2017). The increase in soil RMC content and its significant and positive correlation with the dominant saprotrophic fungal genera advocates the proliferation of saprotrophic fungi driven by the labile C source in response to the SSF amendment. Further, the high Fe content in SSF likely increases Fe reduction in anaerobic environments and can enhance dissolved organic C (easily accessible C source) due to increased pH and colloid dispersion (Buettner et al., 2014), which in turn can increase the population of saprotrophic fungi.

Silicon availability can increase the population of soil fungi. In this study, we found a significant and positive correlation between Si and fungal community. Wainwright et al. (1997) reported that Si compounds can increase fungal growth under both oligotrophic and nutrient-rich conditions. Several possible mechanisms underlying the stimulatory effects of Si on fungal growth have been proposed (Wainwright et al., 1997): (1) silicon compounds or the bioavailable form of Si (i.e., silicic acid) are efficient at absorbing nutrients from the soil, which then acted as nutrient sources for the growth of fungi, (2) silicic acid enhances hyphal growth and stimulates fungal spore germination, and (3) fungi can grow chemoautotrophically by using energy gained from Si metabolism. The molecular mechanism of the stimulatory effects of Si on fungal growth is not known. Notably, some dominant genera (e.g., *Cladosporium*, *Phialemonium*) and species (e.g., *Phialosimplex caninus*, *Metarhizium anisopliae*) of pathotrophic fungi did not change significantly in response to the SSF amendment (Supplementary Tables S4, S5). Nonetheless, Si application has been reported to increase the resistance of plants to pathogenic fungi, probably for its role in induced systematic resistance by increasing the production of stress hormones, or it can interfere with cation cofactors of enzymes influencing plant pathogenesis (Fauteux et al., 2005). The exact mechanism by which Si modulates plant signaling is, however, not known. The relative abundance of symbiotrophic fungal genera was low or negligible, likely due to the flooded conditions of rice paddies (Vallino et al., 2014).

The remarkable increase in saprotrophic fungal communities indicates its potential role in SOM decomposition under SSF fertilization, because, saprotrophic fungi are considered a key regulator of SOM decomposition and can contribute up to 90% of total heterotrophic respiration (Joergensen and Wichern, 2008). Noteworthy, many fungi could also promote soil carbon sequestration through the synthesis of recalcitrant substances and mostly the dominance of mycorrhizal fungal communities suggested an enhanced soil C sequestration (Wilson et al., 2009; Xu et al., 2017), whereas the saprophytic fungal dominance favored C degradation (Joergensen and Wichern, 2008; Wang et al., 2014). In this study, SOC content did not change in response to the short-term SSF amendment. SOC responds slowly to agricultural management practices, which implies that the changes in SOC need many years to be detectable due to the substantial amount of SOC present in soil profile compared to the much smaller proportion of organic C being stored or lost from the soil annually (Haddaway et al., 2015). In comparison to the unamended control, the SSF amendment significantly increased CO_2_ production (SOC mineralization) and the fungal CO_2_ production was increased by 32.0%. Studies conducted in upland arable soil using selective microbial inhibitors indicate that fungi dominate microbial biomass, and CO_2_ derived from the fungal decomposition of SOM dominates the CO_2_ evolved from soils (Joergensen and Wichern, 2008). Flooding the soil favors bacterial proliferation in comparison to the fungal proliferation and may be for this reason, in this study, bacterial contribution to the heterotrophic respiration is more, compared to the fungal respiration. Higher CO_2_ emissions from rice paddies further suggested both heterotrophic and autotrophic respiration increased under SSF fertilization. Saprotrophic fungi degrade SOM as a result of their ability to produce a wide range of extracellular enzymes (Voříšková et al., 2014). Several dominant saprotrophic fungal genera showed significant and positive correlation with both hydrolytic and oxidative enzyme activities and CO_2_ emissions (Figure 8). The SSF amendment significantly increased both soil hydrolytic and oxidase enzyme activities. Soil enzymes are synthesized by microorganisms to degrade specific organic compounds by hydrolysis (hydrolytic enzymes) or oxidation (oxidative enzymes) (Burns et al., 2013). Generally, extracellular enzymes instigating the decomposition of SOM fractions are separated into two groups; hydrolytic enzymes (causing the decomposition of labile organic matter to gain nutrients for primary metabolism) and oxidative enzymes (causing the decomposition of recalcitrant organic matter for co-metabolic acquisition of nutrients) (Nannipieri et al., 2003; Burns et al., 2013). The higher labile carbon availability could increase hydrolytic enzyme activities (de Vries and Caruso, 2016), whereas a greater O_2_ availability, which is due to the higher root oxidase activity could increase oxidase enzyme activities in response to SSF fertilization. Oxygen availability apparently affects oxidase enzyme activities that require O_2_ as a substrate (Hall and Silver, 2013). Moreover, as a rich source of iron, the SSF amendment could increase phenol oxidase activities. There is evidence that ferrous iron increases phenol oxidase activity and organic matter decomposition in waterlogged conditions, probably due to the catalysis of additional OH radical production, promoting the oxidation of phenolics (Van Bodegom et al., 2005). Hall and Silver (2013) reported that amending soils with ferrous iron at field concentrations stimulates short-term C mineralization by up to 27.0%. Hall et al. (2014) also demonstrated that hydrolytic enzyme activities increase with ferrous iron under anaerobic conditions, in contrast to the “enzyme latch” hypothesis. Ferrous iron availability under rhizospheric microaerophilic conditions in rice paddies could play a pivotal role in SOM decomposition since ferrous and ferric iron is the dominant redox couple in submerged rice cropping systems and decomposition of organic matter is a redox driven process (Kögel-Knabner et al., 2010). To achieve a deeper understanding of the role of fungi in SOM decomposition under SSF fertilization, it would be necessary, however, to complement the current data with a functional analysis of metatranscriptomes.

## Conclusion

The slag silicate fertilizer (SSF) markedly increased the relative abundance of saprotrophic fungi, driven by higher labile carbon and Si availability. The SSF also increased soil carbon fluxes, due to increasing hydrolytic and oxidative enzyme activities. Yet, the mechanisms for increasing enzyme activities are different: hydrolytic enzymes were driven by greater labile carbon while oxidative enzymes were driven by root oxidase activity and iron redox cycling. Overall, the SSF amendment alters fungal communities and enzyme activities, and accelerates SOM decomposition, indicating a potential soil carbon loss in the rice fields. Long-term field studies with diverse soil and climate conditions are essential to shed light on the soil microbial behavior and their cascade effects on carbon sequestration under SSF fertilization in rice cropping systems.

## Data Availability Statement

The datasets generated for this study can be found in the BioProject PRJNA491385.

## Author Contributions

SD designed the research, analyzed the data, and wrote the first draft of the manuscript. SD, JL, SC, and HS conducted the experiments. All authors contributed to the intellectual input and assistance to this study and manuscript preparation.

## Conflict of Interest

The authors declare that the research was conducted in the absence of any commercial or financial relationships that could be construed as a potential conflict of interest.
