# Supplementary material for: Silicate Fertilizer Amendment Alters Fungal Communities and Accelerates Soil Organic Matter Decomposition
Source: Front Microbiol. 2019 Dec 20;10:2950. doi: 10.3389/fmicb.2019.02950 (PMC6932956; doi:10.3389/fmicb.2019.02950)
Supplement: Supplementary file 1 [file Table_1.DOCX]

**Supplementary material**

**Silicate fertilizer amendment alters fungal communities and accelerates soil organic matter decomposition**

***Suvendu Das ^1^, Jeong Gu Lee ^2^, Song Rae Cho ^2^, Hyeon Ji Song ^2^, Pil Joo Kim ^1, 2,*^***

*^1^ Institute of Agriculture and Life Sciences, Gyeongsang National University, Jinju, 660-701, Republic of Korea*

*^2^ Division of Applied Life Science, Gyeongsang National University, Jinju, 660-701, Republic of Korea*

^*^Corresponding Author: Pil Joo Kim, E-mail: [pjkim@gnu.ac.kr](mailto:pjkim@gnu.ac.kr)

Phone: +82-55-751-5466; Fax: +82-55-757-0178 14

**Gas sampling and CO_2_ emission estimation**

A closed-chamber method was used to measure CO_2_ emissions from rice planted pots during rice cultivation (Gwon et al., 2018). The gas collection chambers having a diameter of 24 cm and height of 100 cm with a circulating fan for gas mixing and thermometers to monitor inside temperature were placed on bases of rice planted pots during gas sampling. The gas samples were collected from chambers using 50 mL air-tight syringes at 0, 15 and 30 min intervals after chamber placement and transferred into pre-evacuated 20 mL glass vials fitted with butyl rubber stoppers for analysis in the laboratory. Gas sampling was carried out once a week and three times (08:00, 12:00 and 16:00h) in a day to get the average CO_2_  emission flux.

Carbon dioxide concentrations in the collected air samples were measured by gas chromatography (Shimadzu, GC-2010, Japan) packed with a Porapak NQ column (Q 80–100 mesh). Quantification of CO_2_ was done by using a thermal conductivity detector**.** The temperatures of the column, injector, detector and methanizer were adjusted at 80, 100, and 110 and 350 ^o^C respectively. Helium was used as the carrier gas. CO_2_ emission rates were calculated from the increase in gas concentration per unit surface area of the chamber for a specific time interval.

The following equation was used to estimate seasonal CO_2_ fluxes from each treatment (Matsuura et al., 2011).

F = ρ× (V/A) x (Δc /Δt) × (273/T) (1)

where F (mg m^-2^ hr^-1^) is the CO_2_ emission rate, ρ is the gas density of CO_2_ under a standardized state (mg cm^-3^), V is the volume of the chamber (m^3^), A is the surface area of the chamber (m^2^), Δc/Δt is the rate of CO_2_ increase inside the chamber (mg m^-3^ hr^-1^) and T (absolute temperature) is 273 + mean temperature in °C inside the chamber.

The cumulative CO_2_ emissions for the entire cultivation period were calculated using the following equation.

n

i

(Ri × Di)

Cumulative CO_2_ emissions = ∑ (2)

where, R*_i_* was the CO_2_ emissions in the i^th^ sampling intervals, D*_i_* was the number of days in the i^th^ sampling interval, and *n* was the number of sampling intervals.

**CO_2_ production**

The amount of CO_2_ produced under laboratory incubation was calculated using the following formula (Das and Adhya, 2012):

Rate of CO_2_ production (mg kg^-1^ dry soil day^-1^) = (C_HS_ × V_HS_ × ρ) / Ws

Where C_HS_ = concentration of CO_2_ in the headspace (calibrated against standard CO_2_),

V_HS =_ volume of headspace of the bottle,

ρ = density of gas at a given temperature [ρ = (MW × T_ST_) / (MV × T_ST_ + T)

where MW = molecular weight of CO_2_ (44.01 × 10^-3^ mg)

MV = volume at standard conditions (273.2 K) = 22.41 × 10^-3^ m^2^

T = ℃, T_ST_ = 273.2 K, Ws = dry weight of soil

The contribution of fungal or bacterial decomposition (CO_2_ production) was determined in the soil incubation experiment with added streptomycin sulfate (a bactericide) or cycloheximide (a fungicide) (Ananyeva et al., 2010; Seo and DeLaune, 2010). Streptomycin sulfate was added to soils at 3.0 mg g^-1^, while Cycloheximide was added to soils at 1.3 mg g^-1^. Optimal inhibitor concentrations were determined using a preliminary screening experiment based on the criteria of Anderson and Domsch (1975). Optimal inhibitor concentrations were determined using a preliminary screening experiment based on the criteria of Anderson and Domsch (1975). Streptomycin sulfate (0, 1.0, 2.0, 2.5, 3.0, 3.5, 4.0, 5.0, and 10.0 mg g^−1^ soil), and Cycloheximide (0, 1.0, 2.0, 2.5, 3.0, 3.5, 4.0, 5.0, and 10.0 mg g^−1^ soil) were evaluated as inhibitors. To comply with the criteria of selective inhibition, the additive ratios for pairs of inhibitor concentrations was determined. Optimum joint concentrations of inhibitors are indicated when the additive ratio [(A−B)+(A−C)]/(A−D) approached 1; where A is CO_2_ evolved from control only; B is CO_2_ evolved from streptomycin; C is CO_2_ evolved from cycloheximide; and D is CO_2_ evolved from streptomycin+cycloheximide.

**Table S1.** Physicochemical properties of silicate fertilizer

| Parameters | Content |
| --- | --- |
| pH (H_2_O) | 9.6 |
| Fe_2_O_3_ (%) | 5.8 |
| SiO_2_ (%) | 33.6 |
| CaO (%) | 41.5 |
| Organic C (%) | 2.62 |
| Total N (%) | 0.37 |
| P_2_O_5_ (%) | 0.16 |
| Total Fe (%) | 4.93 |
| Total Si (%) | 11.2 |
| Citrate extractable (mg kg^-1^) |  |
| Fe | 9.96 |
| As | 0.24 |
| Hg | 0.13 |
| Cd | Undetectable |
| Pb | Undetectable |

**Table S2.** Statistical analysis of the measured soil physicochemical and plant parameters, CO_2_ emissions and production, and soil enzyme activities as influenced by silicate fertilizer, cultivar and their interactions. Note: Part of the data were published in our earlier paper published in Environment International (Das et al., 2019).

| Parameters | Silicate | Cultivar | Silicate × Cultivar |
| --- | --- | --- | --- |
| pH | 312 ^***^ | 1.65 ^ns^ | 0.33 ^ns^ |
| Eh | 1.05 ^ns^ | 0.13 ^ns^ | 0.005 ^ns^ |
| SOC | 1.72 ^ns^ | 1.11 ^ns^ | 0.83 ^ns^ |
| RMC | 37.4 ^***^ | 7.16 ^*^ | 0.19 ^ns^ |
| NRN | 8.03 ^*^ | 0.97 ^ns^ | 0.004 ^ns^ |
| Root oxidase activity | 13.7 ^**^ | 2.56 ^ns^ | 0.08 ^ns^ |
| Photosynthetic rate | 8.66 ^*^ | 1.48 ^ns^ | 0.04 ^ns^ |
| Root biomass | 7.23 ^*^ | 23.6 ^***^ | 0.22 ^ns^ |
| Straw biomass | 12.9 ^**^ | 23.8 ^***^ | 1.05 ^ns^ |
| Grain yield | 7.52^*^ | 2.23 ^ns^ | 0.09 ^ns^ |
| aqSi | 568 ^***^ | 41.6 ^***^ | 5.28 ^*^ |
| aqFe | 186 ^***^ | 33.8 ^***^ | 5.08 ^*^ |
| CO_2_ emission | 11.3 ^*^ | 1.42 ^ns^ | 0.15 ^ns^ |
| CO_2_ production (no inhibitor) | 8.06 ^*^ | 0.76 ^ns^ | 0.013 ^ns^ |
| CO_2_ production (+Streptomycin) | 8.36 ^*^ | 0.83 ^ns^ | 0.008 ^ns^ |
| CO_2_ production (+Cycloheximide) | 8.44 ^*^ | 0.69 ^ns^ | 0.007 ^ns^ |
| Soil enzyme activities |  |  |  |
| α-glucosidase | 9.04 ^*^ | 0.13 ^ns^ | 0.021 ^ns^ |
| β-1,4-glucosidase | 13.3 ^**^ | 2.26 ^ns^ | 0.14 ^ns^ |
| β-1,4-xylosidase | 8.16 ^*^ | 0.41 ^ns^ | 0.000 ^ns^ |
| Cellobiohydrolase | 8.57 ^*^ | 0.19 ^ns^ | 0.004 ^ns^ |
| Phenol oxidase | 14.8 ^**^ | 0.73 ^ns^ | 0.008 ^ns^ |
| Peroxidase | 12.7 ^**^ | 0.46 ^ns^ | 0.003 ^ns^ |

F value, followed by significant label (*P*) are provised. ^***^, ^**^, ^*^, and ns indicates Significant at *P* < 0.001, at *P* < 0.01, at *P* < 0.05, and non significance, respectively. RMC, readily mineralizable carbon; NRN, Ninhydrin nitrogen, _aq_, water soluble.

**Table S3.** The result of assembly

| Sample Name | Total Bases | Read Count | N (%) | GC (%) | Q20 (%) | Q30 (%) |
| --- | --- | --- | --- | --- | --- | --- |
| IC | 60,766,014 | 151,986 | 0 | 53.24 | 98.58 | 95.21 |
| IS | 61,265,509 | 157,551 | 0 | 52.96 | 98.58 | 95.21 |
| JC | 66,885,087 | 174,372 | 0 | 53.44 | 98.71 | 95.61 |
| JS | 59,823,557 | 151,052 | 0 | 53.21 | 98.66 | 95.43 |

JC, Japonica rice cultivated without SSF; JS, Japonica rice cultivated with SSF; IC, Indica rice cultivated without SSF; IS, Indica rice cultivated with SSF.

**Table S4.** PERMANOVA statistics for slag silicate fertilizer (SSF), cultivar, and SSF × cultivar factors for major fungal (a) phylum, (b) class, (c) order, and (d) genus shown in Figure 5. Letters in bold are significant. Note: Part of the data were published in our earlier paper published in Environment International (Das et al., 2019).

|  | SSF | | Cultivar | | SSF × Cultivar | |
| --- | --- | --- | --- | --- | --- | --- |
|  | *F*-ratio | *P* value | *F*-ratio | *P* value | *F*-ratio | *P* value |
| (a) |  |  |  |  |  |  |
| Ascomycota | **33.6** | **0.000** | 0.82 | 0.46 | 0.18 | 0.82 |
| Basidiomycota | **22.3** | **0.000** | 1.02 | 0.35 | 0.06 | 0.96 |
| Chytridiomycota | **36.4** | **0.000** | 3.16 | 0.13 | 0.05 | 0.98 |
| Glomeromycota | 1.19 | 0.38 | 0.56 | 0.52 | 0.06 | 0.95 |
| Zygomycota | 3.26 | 0.18 | 0.47 | 0.54 | 0.11 | 0.83 |
| (b) |  |  |  |  |  |  |
| Eurotiomycetes | **36.5** | **0.000** | 1.84 | 0.23 | 0.06 | 0.97 |
| Sordariomycetes | **23.6** | **0.000** | 1.56 | 0.25 | 0.36 | 0.67 |
| Agaricomycetes | **9.3** | **0.005** | 1.93 | 0.17 | 0.53 | 0.46 |
| Dothideomycetes | **6.7** | **0.017** | 1.66 | 0.23 | 0.51 | 0.48 |
| Leotiomycetes | 1.22 | **0.31** | 0.55 | 0.51 | 0.08 | 0.93 |
| Chytridiomycetes | **21.4** | **0.000** | 0.83 | 0.45 | 0.15 | 0.82 |
| Incertae sedis | 1.19 | 0.38 | 0.54 | 0.51 | 0.13 | 0.84 |
| Pezizomycetes | 3.33 | 0.18 | 0.47 | 0.56 | 0.11 | 0.85 |
| Tremellomycetes | 1.23 | 0.32 | 2.55 | 0.22 | 0.08 | 0.93 |
| Saccharomycetes | 1.20 | 0.36 | 0.83 | 0.46 | 0.14 | 0.82 |
| (c) |  |  |  |  |  |  |
| Agaricales | **27.4** | **0.000** | 0.85 | 0.43 | 0.20 | 0.78 |
| Sordariales | **44.7** | **0.000** | 1.92 | 0.17 | 0.13 | 0.84 |
| Eurotiales | **38.4** | **0.000** | 1.91 | 0.17 | 0.52 | 0.49 |
| Onygenales | **45.6** | **0.000** | 1.66 | 0.23 | 0.36 | 0.69 |
| Hypocreales | **6.6** | **0.019** | 0.46 | 0.54 | 0.09 | 0.93 |
| Capnodiales | **54.2** | **0.000** | 1.54 | 0.26 | 0.15 | 0.82 |
| Incertae sedis | 2.56 | 0.31 | 0.47 | 0.53 | 0.05 | 0.96 |
| Helotiales | 3.26 | 0.18 | 1.75 | 0.19 | 0.08 | 0.92 |
| Rhizophydiales | **37.3** | **0.000** | 1.93 | 0.16 | 0.53 | 0.46 |
| Mortierellales | **22.8** | **0.000** | 0.82 | 0.47 | 0.14 | 0.85 |
| Pleosporales | 1.23 | 0.33 | 0.55 | 0.52 | 0.15 | 0.85 |
| Saccharomycetales | 1.19 | 0.37 | 0.52 | 0.53 | 0.36 | 0.69 |
| (d) |  |  |  |  |  |  |
| Chrysosporium | **54.6** | **0.000** | 1.78 | 0.21 | 0.48 | 0.56 |
| Rhizophydium | **21.2** | **0.000** | 1.11 | 0.37 | 0.14 | 0.84 |
| Mortierella | **43.6** | **0.000** | 1.92 | 0.18 | 0.22 | 0.75 |
| Phialosimplex | **53.7** | **0.000** | 3.16 | 0.12 | 0.46 | 0.57 |
| Acremonium | **46.7** | **0.000** | 1.93 | 0.17 | 0.53 | 0.47 |
| Geomyces | **7.9** | **0.008** | 1.26 | 0.92 | 0.44 | 0.59 |
| Talaromyces | **6.8** | **0.017** | 1.74 | 0.19 | 0.08 | 0.92 |
| Cladosporium | 3.33 | 0.19 | 0.43 | 0.57 | 0.13 | 0.88 |
| Arthrobotrys | **9.1** | **0.005** | 2.75 | 0.19 | 0.17 | 0.83 |
| Phialemonium | 2.85 | 0.26 | 0.85 | 0.48 | 0.11 | 0.84 |
| Williopsis | **6.7** | **0.019** | 1.26 | 0.92 | 0.27 | 0.64 |
| Penicillium | 1.81 | 0.29 | 0.52 | 0.51 | 0.13 | 0.84 |

**Table S5.** The guild of some dominant fungal species

| Fungal species | Relative abundance (%) | | | | Guild | Reference |
| --- | --- | --- | --- | --- | --- | --- |
|  | JC | JS | IC | IS |  |  |
| *Chrysosporium keratinophilum* | 1.03 | 1.82 | 0.49 | 2.07 | Saprotroph | Bohacz and Kornillowicz-Kowalska, 2012 |
| *Mortierella polycephala* | 0.64 | 1.46 | 0.52 | 1.77 | Saprotroph | Domsch et al., 1980 |
| *Agaricales sp* | 1.05 | 1.53 | 0.81 | 1.72 | Saprotroph | Rosa and Capelari, 2009 |
| *Helotiales* sp. | 0.38 | 1.06 | 0.31 | 1.52 | Saprotroph | Newsham et al., 2018 |
| *Phialemonium inflatum* | 0.26 | 0.81 | 0.28 | 0.72 | Saprotroph | Perdomo et al., 2013 |
| *Aspergillus sydowii* | 0.11 | 0.85 | 0.13 | 0.72 | Saprotroph | Cai and Lu, 2012 |
| *Funneliformis mosseae* | 0.07 | 0.13 | 0.06 | 0.19 | Symbiotroph | Gui et al., 2017 |
| *Metarhizium anisopliae* | 0.04 | 0.08 | 0.07 | 0.06 | Pathotrophs | Cloyd 1999 |

**Table S6.** Correlation coefficient (*r*) between soil variables and fungal community determined by Mantel test^†^

|  | *r* | *P* |
| --- | --- | --- |
| RMC | 0.293 | **0.001** |
| NRN | 0.024 | 0.375 |
| _aq_Si | 0.203 | **0.010** |
| _aq_Fe | 0.168 | **0.031** |
| Soil pH | 0.152 | **0.044** |
| Soil Eh | -0.009 | 0.134 |
| Root biomass | 0.089 | 0.137 |
| Shoot biomass | 0.043 | 0.287 |
| Root oxidase activity | 0.114 | **0.054** |
| Photosynthetic rate | 0.097 | 0.123 |
|  |  |  |
| α-glucosidase | 0.198 | **0.018** |
| β-1,4-glucosidase | 0.267 | **0.001** |
| β-1,4-xylosidase | 0.195 | **0.018** |
| Cellobiohydrolase | 0.156 | **0.043** |
| Phenol oxidase | 0.246 | **0.001** |
| Peroxidase | 0.201 | **0.010** |
| CO_2_ emission | 0.151 | **0.045** |
| Fungal CO_2_ production | 0.155 | **0.043** |

^††^ Permutations, 9,999 (using the relative abundances of OTU as input in the analysis).

The significant values (*P* < 0.10) are indicated in bold. RMC, readily mineralizable carbon; NRN, Ninhydrin nitrogen.


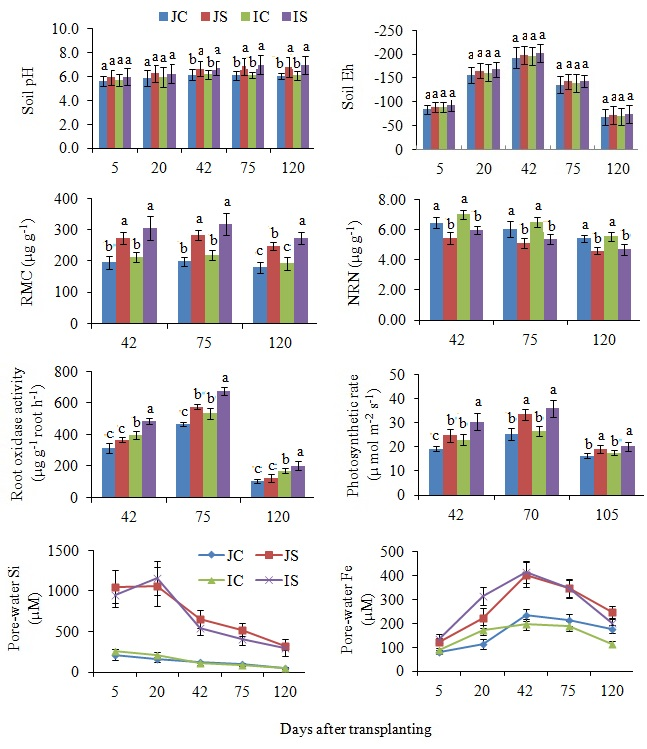


**Figure S1.** Effects of slag silicate fertilizer (SSF) amendment on soil biochemical properties, photosynthetic rate, and porewater Si and Fe concentration in Japonica and Indica rice cultivars. Bars represent standard deviation. Different lower case letters represent a significant difference at *P*< 0.05. JC, Japonica rice cultivated without SSF; JS, Japonica rice cultivated with SSF; IC, Indica rice cultivated without SSF; IS, Indica rice cultivated with SSF.

**
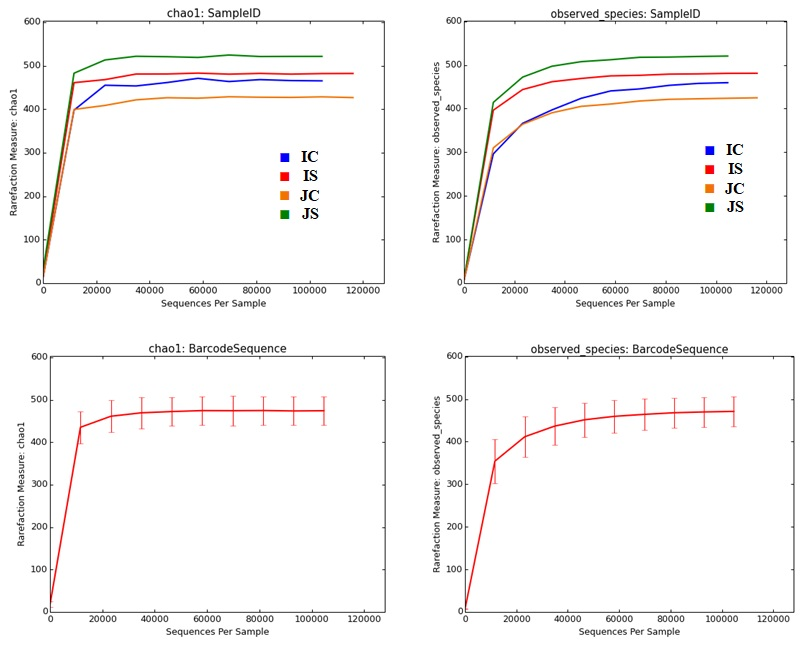
**

**Figure S2.** . Rarefaction curves of Chao1and the observed species based on treatment (samples) and barcode sequences. JC, Japonica rice cultivated without SSF; JS, Japonica rice cultivated with SSF; IC, Indica rice cultivated without SSF; IS, Indica rice cultivated with SSF.

**References**

Ananyeva, N.D., Stolnikova, E.V., Susyan, E.A., Khodzhaeva, A.K., 2010. The fungal and bacterial biomass (selective inhibition) and the production of CO_2_ and N_2_O by Soddy Podzolic soils of postagrogenic biogeocenoses. Eurasian Soil Science 43(11), 1287–1293.

Anderson, J.P.E., Domsch, K.H., 1975. Measurement of bacterial and fungal contributions to respiration of selected agricultural and forest soils. Canadian Journal of Microbiology 21, 314-322.

Bohacz, J., Korniłłowicz-Kowalska, T., 2012. Species diversity of keratinophilic fungi in various soil types. Central European Journal of Biology 7(2), 259-266.

Cai, G.L., Lu, J., 2012. Isolation and identification of a novel Aspergillus sydowii F5 Producing alpha-galactosidase and statistical optimization for the enzyme production. Asian Journal of Chemistry 24(2), 541–5.

Cloyd, R.A., 1999. [The entomopathogenic fungus *Metarhizium anisopliae*](http://www.entomology.wisc.edu/mbcn/kyf607.html). Midwest Biological Control News. VI (7).

Das, S., Adhya, T.K., 2012. Dynamics of methanogenesis and methanotrophy in tropical paddy soils as influenced by elevated CO_2_ and temperature interaction. Soil Biology & Biochemistry 47, 36 - 45.

Das, S., Gwon, H.S., Khan, M.I., Van Nostrand, J.D., Alam, M.A., and Kim, P. J. 2019. Taxonomic and functional responses of soil microbial communities to slag-based fertilizer amendment in rice cropping systems. Environment International, 127, 531-539.

Domsch, K.H., Gams, W., Andersen, T.H., 1980. Compendium of soil fungi (2nd ed.). London, UK: Academic Press. [ISBN](https://en.wikipedia.org/wiki/International_Standard_Book_Number) [978-0122204012](https://en.wikipedia.org/wiki/Special:BookSources/978-0122204012).

Gui, H., Purahong, W., Hyde, K.D., Xu, J., Mortimer, P.E., 2017. The Arbuscular Mycorrhizal Fungus Funneliformis mosseae Alters Bacterial Communities in Subtropical Forest Soils during Litter Decomposition. Frontiers in Microbiology 8, 1120.

Gwon, H.S., Khan, M.I., Alam, M.A., Das, S., Kim, P.J., 2018. Environmental risk assessment of steel-making slags and the potential use of LD slag in mitigating methane emissions and the grain arsenic level in rice (*Oryza sativa* L.). Journal of Hazardous Materials 353, 236–243.

Matsuura, S., Mori, A., Hojito, M., Kanno, T., Sasaki, H., 2011. Evaluation of a portable chamber system for soil CO_2_ efflux measurement and the potential errors caused by internal compensation and water vapor dilution. Journal of Agricultural Meteorology 67, 127-137.

Newsham, K.K., Garnett, M.H., Robinson, C.H., Cox, F., 2018. Discrete taxa of saprotrophic fungi respire diferent ages of carbon from Antarctic soils. Scientific Reports 8, 7866.

[Perdomo, H., García, D., Gené, J., Cano, J., Sutton, D.A., Summerbell, R., Guarro, J., 2013. Phialemoniopsis, a new genus of Sordariomycetes, and new species of Phialemonium and Lecythophora. Mycologia. 105(2), 398-421](http://www.westerdijkinstitute.nl/Indoor/BioloMICS.aspx?TableKey=14682616000000061&Rec=55713&Fields=All).

Rosa, L.H., Capelari, M., 2009. Agaricales fungi from atlantic rain forest fragments in minas gerais, Brazil. Brazilian Journal of Microbiology 40, 846-851.

Seo, D.C., DeLaune, R.D., 2010. Effect of redox conditions on bacterial and fungal biomass and carbon dioxide production in Louisiana coastal swamp forest sediment. Science of the Total Environment 408, 3623–3631.
